# Missed radiation therapy sessions in first three weeks predict distant metastasis and less favorable outcomes in surgically treated patients with oral cavity squamous cell carcinoma

**DOI:** 10.1186/s13014-020-01632-1

**Published:** 2020-08-14

**Authors:** Yin-Yin Chiang, Yung-Chih Chou, Kai-Ping Chang, Chun-Ta Liao, Yao-Yu Wu, Wing-Keen Yap, Ping-Ching Pai, Joseph Tung-Chieh Chang, Chien-Yu Lin, Kang-Hsing Fan, Bing-Shen Huang, Tsung-Min Hung, Ngan-Ming Tsang

**Affiliations:** 1 Department of Radiation Oncology, Proton and Radiation Therapy Center, Linkou Chang Gung Memorial Hospital and Chang Gung University, Taoyuan City, Taiwan; 2 Department of Otolaryngology-Head Neck Surgery, Linkou Chang Gung Memorial Hospital and Chang Gung University, Taoyuan City, Taiwan; 3 College of Medicine Chang Gung University, Taoyuan, Taiwan; 4 Department of Head and Neck Oncology, Linkou Chang Gung Memorial Hospital and Chang Gung University, Taoyuan, Taiwan; 5 School of Traditional Chinese Medicine, Chang Gung University, Taoyuan, Taiwan; 6 Department of Radiation Oncology, Fangliao General Hospital, No. 139, Zhongshan Rd., Fangliao Township, Pingtung County 940 Taiwan; 7 Department of Radiation Oncology, Chang Gung Memorial Hospital at Lin-Kou, School of Traditional Chinese Medicine, Chang Gung University, No. 5 Fu-Hsing Street, Kwei-Shan, Taoyuan, 333 Taiwan

**Keywords:** Oral cavity cancer, Squamous cell carcinoma, Radiation therapy, Missed sessions, Overall survival, Distant metastasis, Prognosis

## Abstract

**Background:**

We sought to investigate the prognostic impact of missed RT sessions in patients who had undergone surgery for oral cavity squamous cell carcinoma (OCSCC).

**Methods:**

The study sample consisted of 905 patients with surgically treated OCSCC who fulfilled criteria of RT course ≤8 weeks. The study participants were divided into three groups based on the characteristics of missed RT, as follows: 1) early missed RT, 2) late missed RT, and 3) RT as scheduled.

**Results:**

The 5-year overall survival (OS) rates in the early missed RT, late missed RT, and RT as scheduled groups were 53.0, 58.1, and 64.5%, respectively (*p* = 0.046). In multivariate analysis, early missed RT was independently associated with both OS (hazard ratio (HR) = 1.486; 95% confidence interval (CI): 1.122–1.966; *p* = 0.006) and the occurrence of distant metastasis (HR = 1.644; 95% CI: 1.047–2.583; *p* = 0.031).

**Conclusion:**

Early missed RT was independently associated with a higher occurrence of distant metastasis and less favorable OS in patients who had undergone surgery for OCSCC.

## Background

Malignancies of the oral cavity represent a major public health concern, with over 350,000 new yearly cases being diagnosed worldwide [1]. In Taiwan, oral cavity cancer accounts for approximately 70% of all newly diagnosed head and neck tumors [2] – with oral cavity squamous cell carcinoma (OCSCC) being the most common histological type. Radical surgery remains the mainstay of treatment for OCSCC. Patients carrying unfavorable pathological risk factors are also candidate to receive adjuvant radiotherapy (RT) – either with or without chemotherapy – in an effort to improve local and systemic control rates [3–6]. Adherence to the initially prescribed RT regimen is paramount to ensure optimal treatment outcomes, and deviations from the initial schedule may have adverse prognostic implications. In this context, published studies have shown that several variables related to the RT schedule – including length of radiation time, premature discontinuation of RT, and time to initiation of postoperative RT – predict prognosis in patients with head and neck malignancies [7–12].

The question as to whether missed RT sessions could be associated with clinical outcomes has received less attention [7], even in studies that focused on RT as definitive treatment in patients who did not undergo radical surgery [13–15]. We therefore designed the current retrospective study to specifically investigate this issue in a large series of patients with OCSCC who had previously undergone surgery. Missed sessions were analyzed in relation to their temporal occurrence during the RT course and examined with respect to overall survival (OS) and other outcomes of interest – including locoregional control (LRC) and freedom from distant metastasis (FFDM) rates.

## Methods

### Study patients

We retrospectively reviewed the clinical records of 1058 adult patients with histology-proven OCSCC who received radical surgery and RT – either alone or in combination with chemotherapy – at the Linkou Chang Gung Memorial Hospital (Taoyuan City, Taiwan) between January 2005 and December 2012. All participants were restaged according to the American Joint Committee on Cancer (AJCC) Staging Manual, Eighth Edition by an experienced radiation oncologist after reviewing medical records. The institutional tumor board performed central review of each new case. Before the routine use of positron emission tomography (PET) imaging (2009), staging was performed with computed tomography (CT) or magnetic resonance (MR) imaging along with abdominal ultrasound and bone scan. Currently, both MR and PET can be performed for staging purposes. As of 2009, tumor board routinely suggested a second PET scan (post-operative/pre-RT PET) prior to adjuvant treatment for high-risk patients (e.g., those presenting with extranodal spread) to detect early recurrences. Only patients who underwent surgery with curative intent (including neck dissection) and who received a radiation dose of 2 Gy per fraction were deemed eligible. Exclusion criteria were as follows: 1) history of second primary malignancies; 2) local, regional, or distant failure occurring during the course of RT; 3) total RT dose < 60 Gy or > 72 Gy; and 4) presence of distant recurrences identified on the second PET scan. Owing to the retrospective nature of the study, the need for informed consent was waived.

### Treatment approach

Primary tumors were excised with ≥1 cm margins (both peripheral and deep margins). Patients with clinically positive nodal disease underwent level I–V neck dissections, whereas level I–III neck dissections were performed in presence of clinically negative nodes. The procedures for collection and classification of pathologic risk factors and the indications for adjuvant treatment have been previously described in detail [16, 17]. Patients received homogeneous treatment according to our institutional guidelines (Supplementary Table 1). All patients underwent postoperative radiotherapy (PORT) consisting of a conventional fractionated daily dose of 2 Gy per fraction, 5 days per week. A 6-MV photon beam was used to achieve a target total dose between 60 and 66 Gy. Suspicious FDG-avid lesions detected on PET imaging before the beginning of RT received a simultaneous integrated boost at a dose of 70–72 Gy [18]. The initial treatment volume comprised the primary tumor bed and the regional cervical nodes. PORT was performed using different techniques – including conventional two-dimensional RT, three-dimensional conformal RT, intensity-modulated radiation therapy (IMRT), and volumetric-modulated arc therapy (VMAT). A conventional field arrangement generally included a bilateral opposing field and a low anterior portal – with the spinal cord being shielded upon delivering of a dose of 46 Gy or more. The dose delivered to the brain stem and spinal cord was limited to 54 Gy. Upon administration of 46–50 Gy, the irradiation area was reduced to include the tumor bed and metastatic nodes only. Concurrent chemotherapy was offered to patients harboring adverse prognostic factors [17]. Cisplatin – generally administered as a single dose of 100 mg/m^2^ every 3 weeks or at a weekly dose of 40 mg/m^2^ – was the most commonly used chemotherapy agent [19, 20].

### Definition of variables

The performance status was calculated with the Eastern Cooperative Oncology Group scale. Cigarette smoking was dichotomized as yes (subjects who smoked ≥100 cigarettes in their lifetime) versus no (subjects who smoked < 100 cigarettes in their lifetime and who were not currently smoking) [21]. Alcohol consumption (current or former drinkers versus nondrinkers) and betel quid chewing (current or former chewers versus non-chewers) were also considered as dichotomous variables. Chemotherapy was dichotomized as yes (concurrent chemotherapy or chemotherapy administered in the 2 weeks preceding the start of RT) versus no. The Charlson Comorbidity Index [22] was used to categorize the presence of comorbidities as yes (score ≥ 1) versus no. Preoperative PET imaging was dichotomized as yes versus no.

### Radiotherapy-related variables

Because a prolonged RT course is known to be associated with a less favorable OS [23], we solely focused on patients with a radiation treatment time (RTT) – defined as the time from the beginning to the end of RT – of less than 8 weeks. The treatment package time (TPT) was calculated from the date of surgery to the last RT session. The interval between surgery and RT was calculated from the date of surgery to the first RT session. For the purpose of analysis, the study participants were divided into three groups based on the characteristics of missed RT, as follows: 1) early missed RT (defined as having received < 14 fractions in the first 3 weeks), 2) late missed RT (defined as having received ≥14 fractions in the first 3 weeks but less than 29 fractions in the first 6 weeks), and 3) RT as scheduled (defined as having received ≥14 fractions in the first 3 weeks and ≥ 29 fractions in the first 6 weeks).

### Definition of outcomes

OS – calculated as the time elapsed (in years) from the start of RT to the date of death – was the main outcome measure. Secondary outcomes included LRC and FFDM rates. LRC was defined as the time from the start of RT to the date of local or regional recurrence, whereas FFDM was the time elapsed from the start of RT to the date of diagnosis of distant metastases.

### Data analysis

Intergroup differences in terms of continuous variables were assessed with the Student’s *t*-test, whereas the chi-square test was used for categorical data. Survival curves were plotted with the Kaplan-Meier method (log-rank test). Multivariate Cox proportional hazards regression analyses were used to identify independent predictors of OS, LRC, and FFDM rates. The following covariates were entered into the multivariate model: age, sex, pT, pN, tumor differentiation, TPT, pattern of missed RT sessions (early missed RT, late missed RT, RT as scheduled), cigarette smoking, betel quid chewing, alcohol consumption, presence of comorbidities, concurrent chemotherapy, and PET imaging. The results are expressed as hazard ratios (HRs) with 95% confidence intervals (CIs). In all analyses, two-tailed *p* values < 0.05 were considered statistically significant.

## Results

### Patient characteristics

The median age of the 905 study participants was 50.8 years (range, 25.1–89.4 years) (Table 1). There were 37 (3.1%) patients who had pathological stage I − II disease, whereas 868 (95.9%) had stage III − IV disease. Pathological nodal metastases were identified in 409 patients (63.3%). PET/CT imaging was performed during the preoperative staging work-up in 442 (48.8%) study participants. Concurrent chemoradiation was administered to 505 (55.8%) patients. A total of 642 (70.9%) patients began PORT within 6 weeks of radical surgery, with 436 (48.2%) having a TPT ≤85 days. The median RTT was 47 days (range, 39–56 days). A total of 556 (61.4%) and 905 (100%) patients completed RT within 7 and 8 weeks of surgery, respectively.

**Table 1:** General characteristics of the study patients

	RT as scheduled	Late missed RT	Early missed RT	Entire cohort	*p* value
**Number**	448	341	116	905	
**Sex, n (%)**					0.210
Women	26 (5.8%)	21 (6.2%)	12 (10.3%)	59 (6.5%)	
Men	422 (94.2%)	320 (93.8%)	104 (89.7%)	846 (93.5%)	
**Age (years), median (range)**	50.8 (25.1–89.4)	51.1 (28.0–83.6)	49.8 (25.1–78.8)	50.8 (25.1–89.4)	0.330
**Age (years), n (%)**					0.481
< 60	363 (81.0%)	274 (80.4%)	95 (81.9%)	732 (80.9%)	
≥ 60	85 (19.0%)	67 (19.6%)	21 (18.1%)	173 (19.1%)	
**Differentiation**					0.305
Well differentiated	104 (23.2%)	87 (25.5%)	40 (34.5%)	232 (25.6%)	
Moderately differentiated	284 (63.4%)	199 (58.4%)	62 (53.5%)	545 (60.2%)	
Poorly differentiated	60 (13.4%)	55 (16.1%)	14 (12.0%)	128 (14.2%)	
**pStage, n (%)**					0.710
Stage I	6 (1.3%)	2 (0.6%)	0 (0%)	8 (0.9%)	
Stage II	16 (3.6%)	11 (3.2%)	2 (1.7%)	29 (3.2%)	
Stage III	123 (27.5%)	100 (29.3%)	33 (28.4%)	256 (28.3%)	
Stage IV	303 (67.6%)	228 (66.9%)	81 (69.8%)	612 (67.6%)	
**pT − Stage, n (%)**					0.824
T1/2	58 (12.9%)	40 (11.7%)	16 (13.8%)	114 (12.6%)	
T3/4	390 (87.1%)	301 (88.3%)	100 (86.2%)	791 (87.4%)	
**pN − Stage, n (%)**					0.828
N_0/1_	255 (56.9%)	198 (58.0%)	63 (54.3%)	516 (57.0%)	
N_2/3_	193 (43.1%)	143 (42.0%)	53 (45.7%)	389 (43.0%)	
**RTT (days), median (range)**	46 (39–53)	49 (44–56)	50 (43–56)	47 (39–56)	0.131
Mean ± SD	45.6 ± 2.52	49.0 ± 2.77	50.3 ± 2.94	47.5 ± 3.28	0.120
**S/RT interval (days), median (range)**	39 (15–106)	40 (15–94)	37 (17–77)	39 (15–106)	0.078
Mean ± SD	40.2 ± 12.2	41.6 ± 13.4	38.4 ± 10.4	40.5 ± 12.5	0.061
**S/RT interval (days), n (%)**					0.256
≤ 42	326 (72.8%)	231 (67.7%)	85 (73.3%)	642 (70.9%)	
> 42	122 (27.2%)	110 (32.3%)	31 (26.7%)	263 (29.1%)	
**TPT (days), median (range)**	84 (50–152)	88 (63–143)	86 (65–128)	86 (59–152)	0.058
Mean ± SD	85.8 ± 12.4	90.5 ± 13.8	88.6 ± 10.7	88.0 ± 12.9	0.029
**TPT (days), n (%)**					< 0.001
≤ 85	254 (56.7%)	133 (39.0%)	49 (42.2%)	436 (48.2%)	
> 85	194 (43.3%)	208 (61.0%)	67 (57.7%)	469 (51.8%)	
**RT techniques**					0.691
2D RT	76 (16.9%)	68 (19.9%)	20 (17.2%)	164 (18.1%)	
3D conformal RT	43 (9.6%)	14 (4.1%)	12 (10.3%)	69 (7.6%)	
IMRT	296 (66.1%)	223 (65.4%)	80 (70.0%)	599 (66.2%)	
VMAT	33 (7.4%)	36 (10.6%)	4 (3.5%)	73 (8.1%)	
**Chemotherapy, n (%)**					0.933
Yes	248 (55.4%)	190 (55.7%)	67 (57.8%)	505 (55.8%)	
No	200 (44.6%)	151 (44.3%)	49 (42.2%)	400 (44.2%)	
**PET imaging, n (%)**					0.095
Yes	233 (52.0%)	151 (44.3%)	58 (50.0%)	442 (48.8%)	
No	215 (48.0%)	190 (55.7%)	58 (50.0%)	463 (51.2%)	
**Smoking, n (%)**					0.864
No	65 (14.5%)	46 (13.5%)	18 (15.5%)	129 (14.3%)	
Yes	383 (85.5%)	295 (86.5%)	98 (84.5%)	776 (85.7%)	
**Betel quid chewing, n (%)**					0.366
No	94 (21.0%)	85 (24.9%)	29 (25.0%)	208 (23.0%)	
Yes	354 (79.0%)	256 (75.1%)	87 (75.0%)	697 (77.0%)	
**Alcohol use, n (%)**					0.171
No	182 (40.6%)	116 (34.0%)	43 (37.1%)	341 (37.7%)	
Yes	266 (59.4%)	225 (66.0%)	73 (62.9%)	564 (62.3%)	
**Comorbidities, n (%)**					0.227
No	237 (52.9%)	159 (46.6%)	61 (52.6%)	456 (50.3%)	
Yes	211 (47.1%)	182 (53.4%)	55 (47.4%)	448 (49.5%)	

Data are given as counts (percentages), means ± standard deviations, or medians (ranges), as appropriate. *Abbreviations: RT* radiotherapy, *RTT* radiation treatment time, *S* surgery, *SD* standard deviation, *TPT* treatment package time, *2D* two-dimensional, *3D* three-dimensional, *IMRT* intensity-modulated radiation therapy, *VMAT* volumetric-modulated arc therapy, *PET* positron emission tomography

### Missed radiation therapy sessions

There were 116, 341, and 448 patients in the early missed RT, late missed RT, and RT as scheduled groups, respectively. The reasons for early missed RT were as follows: operational causes (machine breakdown, public holidays; *n* = 69), treatment plan modifications because of physician’s decision (including re-simulation and re-optimization of the treatment plan due to modifications of the facial profile, physician’s discretion, missing of fixation cast or oral bite block; *n* = 13), patients taking leaves (unknown reasons; *n* = 34), frailty (hospital admissions, weight loss; *n* = 18), and mixed reasons (operational causes and other reasons; *n* = 32).

### Survival outcomes

**Fig. 1:**
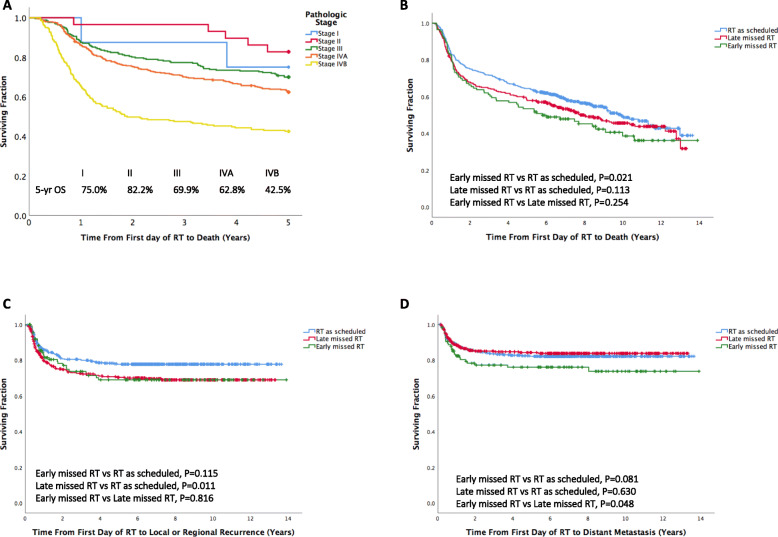
Panel **a**: Kaplan-Meier plot of 5-year overall survival according to pathologic stage (AJCC Eight Edition Staging Manual; *n* = 905); panel **b**: Kaplan-Meier plot of overall survival in patients stratified into three groups based on the characteristics of missed radiation therapy; panel **c**: Kaplan-Meier plot of local-regional control in the study patients stratified into three groups based on the characteristics of missed radiation therapy; panel **d**: Kaplan-Meier plot of freedom from distant metastases in the study patients stratified into three groups based on the characteristics of missed radiation therapy

The median duration of follow-up was 6.1 years (range, 0.2–13.9 years), during which 451 (49.8%) patients died. The 2-, 3-, and 5-year OS rates in the entire study cohort were 71.1, 67.6, and 60.6%, respectively. The 5-year OS rates according to pathologic stage (AJCC Eight Edition Staging Manual) are shown in Fig. 1a. Kaplan-Meier survival plots of OS, FFDM, and LRCR according to the patterns of missed RT sessions (early missed RT, late missed RT, RT as scheduled) are depicted in Fig. 1b−d. The 5-year OS rates in patients with early missed RT, late missed RT, and RT as scheduled were 53.0, 58.1, and 64.5%, respectively. The 5-year OS rate of patients with early missed RT was significantly lower than of patients with RT as schedule (*p* = 0.021). The 5-year FFDM rates in patients with early missed RT, late missed RT, and RT as scheduled were 76.1, 84.3, and 82.2%, respectively. Patients with early missed RT had a significantly higher rate of distant metastasis when compared with patients with late missed RT (*p* = 0.048). Finally, the 5-year LRC rates in patients with early missed RT, late missed RT, and RT as scheduled were 70.1, 69.1, and 77.8%, respectively. Patients with late missed RT had a significantly lower local-regional control rate when compared with patients with RT as scheduled (*p* = 0.011).

### Predictors of survival outcomes

Table 2 summarizes the results of univariate analysis of variables associated with 5-year OS, FFDM, and LRCR rates. In addition to other variables, early missed RT (versus RT as scheduled) was identified as a significant adverse predictor of OS. Moreover, early missed RT (versus late missed RT) was significantly associated with a less favorable FFDM. After allowance for potential confounders in multivariate analysis (Table 3), early missed RT (versus RT as scheduled) retained its independent adverse prognostic significance for OS (HR = 1.201; 95% CI: 0.978–1.474; *p* = 0.008). Notably, early missed RT (versus RT as scheduled) was also independently associated with a less favorable FFDM (HR = 1.644; 95% CI: 1.047–2.583; *p* = 0.031).

**Table 2:** Univariate analyses of freedom from distant metastases, locoregional control, and overall survival rates

	FFDM		LRC		OS	
Covariate	HR (95% CI)	*p* value	HR (95% CI)	*p* value	HR (95% CI)	*p* value
**RT schedule**						
Early missed RT vs. RT as scheduled	1.470 (0.946–2.283)	0.086	1.383 (0.920–2.079)	0.118	1.385 (1.052–1.821)	0.020*
Late missed RT vs. RT as scheduled	0.916 (0.641–1.311)	0.632	1.453 (1.091–1.935)	0.011	1.177 (0.962–1.439)	0.112
Early missed RT vs. late missed RT	1.602 (1.004–2.564)	0.048	1.050 (0.700–1.576)	0.812	1.176 (0.889–1.557)	0.254
**pT Stage (3/4 vs.1/2)**	1.679 (0.951–2.964)	0.074	1.670 (1.043–2.67)	0.033	1.459 (1.072–1.98)	0.016*
**pN Stage (2/3 vs. 0/1)**	3.332 (2.370–4.685)	< 0.001	1.621 (1.243–2.11)	< 0.001	1.708 (1.419–2.05)	< 0.001*
**Differentiation**						
(Moderately vs. Well)	1.647 (1.067–2.541)	0.024	1.264 (0.915–1.747)	0.155	1.215 (0.973–1.518)	0.086
(Poorly vs. Well)	2.395 (1.422–4.033)	0.001	1.201 (0.764–1.890)	0.427	1.224 (0.895–1.674)	0.205
**Sex** (Male vs. Female)	1.169 (0.596–2.292)	0.650	0.901 (0.541–1.49)	0.688	1.110 (0.752–1.63)	0.600
**Age** (> 60 vs. ≤60 years)	0.654 (0.654–1.237)	0.899	0.835 (0.639–1.090)	0.184	1.342 (1.114–1.616)	0.002*
**Chemotherapy** (Yes vs. no)	0.509 (0.361–0.717)	< 0.001	0.742 (0.566–0.974)	0.032	0.718 (0.594–0.867)	< 0.001*
**TPT** (days) (> 85 vs. ≤85)	1.083 (0.788–1.490)	0.622	1.395 (1.038–1.734)	0.026	1.142 (0.948–1.313)	0.161
**Alcohol** (Yes vs. No)	1.291(0.921–1.811)	0.139	1.363 (1.026–1.81)	0.033	1.315 (1.078–1.60)	0.007*
**Betel quid** (Yes vs. no)	1.456 (0.961–2.208)	0.077	1.137 (0.823–1.570)	0.435	1.150 (0.919–1.432)	0.222
**Smoking** (Yes vs. no)	1.421 (0.846–2.385)	0.184	0.923 (0.637–1.336)	0.670	0.978 (0.752–1.2)	0.871
**Comorbidity** (Yes vs. no)	0.953 (0.839–1.585)	0.380	1.047 (0.803–1.366)	0.733	1.210 (1.006–1.456)	0.040*
**S/RT interval**	1.006 (0.708–1.428)	0.975	1.575 (1.142–2.172)	0.006	1.252(1.030–1.521)	0.024*
**PET** (Yes vs. no)	0.838 (0.610–1.153)	0.278	1.522 (1.161–1.997)	0.002	1.119 (0.928–1.350)	0.238

*Abbreviations: FFDM* freedom from distant metastases, *LRC* locoregional control, *OS* overall survival, *RT* radiotherapy, *TPT* total package time, *S* surgery, *PET* positron emission tomography, *HR* hazard ratio, *CI* confidence interval. *Denotes statistically significant *p* values

**Table 3:** Multivariate analyses of freedom from distant metastases, locoregional control, and overall survival rates

	FFDM		LRCR		OS	
Covariate	HR (95% CI)	*p* value	HR (95% CI)	*p* value	HR (95% CI)	*p* value
**RT schedule**						
Early missed RT vs. RT as scheduled	1.644 (1.047–2.583)	0.031	1.422 (0.877–2.204)	0.152	1.486 (1.122–1.966)	0.006*
Late missed RT vs. RT as scheduled	1.022 (0.710–1.472)	0.903	1.389 (1.032–1.878)	0.031	1.201 (0.978–1.474)	0.080
Early missed RT vs. late missed RT	1.683 (1.047–2.702)	0.031	0.981 (0.608–1.582)	0.937	1.246 (0.939–1.652)	0.164
**pT Stage** (3/4 vs.1/2)	1.952 (1.097–3.472)	0.023	1.722 (1.083–2.930)	0.039	1.56 (1.144–2.142)	0.006*
**pN Stage** (2/3 vs.0/1)	3.612 (2.327–5.609)	< 0.001	1.650 (1.170–2.313)	0.007	1.805 (1.423–2.316)	< 0.001*
**Differentiation**						
(Moderately vs. Well)	1.323 (0.844–2.074)	0.223	1.203 (0.859–1.686)	0.282	1.163 (0.921–1.468)	0.205
(Poorly vs. Well)	2.078 (1.204–3.586)	0.009	1.103 (0.690–1.764)	0.682	1.155 (0.825–1.585)	0.389
**Sex** (Male vs. Female)	1.078 (0.463–2.036)	0.938	0.924 (0.530–1.613)	0.781	1.079 (0.746–1.727)	0.726
**Age** (> 60 vs. ≤60 years)	1.082 (0.774–1.513)	0.643	0.884 (0.668–1.170)	0.390	1.500 (1.234–1.824)	< 0.001*
**Chemotherapy** (Yes vs. no)	1.244 (0.798–1.923)	0.335	0.950 (0.696–1.407)	0.955	1.026 (0.791–1.303)	0.915
**TPT** (days) (> 85 vs. ≤85)	0.901 (0.597–1.360)	0.619	1.108 (0.775–1.584)	0.575	0.866 (0.670–1.119)	0.272
**Alcohol** (Yes vs. No)	1.135 (0.789–1.631)	0.496	1.224 (0.901–1.664)	0.197	1.269 (1.025–1.569)	0.028*
**Betel quid** (Yes vs. no)	1.406 (0.897–2.203)	0.137	1.445 (0.926–2.256)	0.105	1.168 (0.913–1.494)	0.216
**Smoking** (Yes vs. no)	1.484 (0.841–2.619)	0.173	0.859 (0.572–1.290)	0.463	0.916 (0.684–1.226)	0.555
**Comorbidity** (Yes vs. no)	1.090 (0.784–1.515)	0.609	1.040 (0.792–1.367)	0.777	1.115 (0.922–1.348)	0.260
**S/RT interval**	1.049 (0.659–1.669)	0.841	1.236 (0.800–1.910)	0.340	1.199 (0.913–1.575)	0.191
**PET** (Yes vs. no)	0.937 (0.665–1.319)	0.708	1.470 (1.048–1.063)	0.026	1.115 (0.922–1.348)	0.138

*Abbreviations: FFDM* freedom from distant metastases, *LRC* locoregional control, *OS* overall survival, *RT* radiotherapy, *TPT* total package time, *S* surgery, *PET* positron emission tomography, *HR* hazard ratio, *CI* confidence interval. *Denotes statistically significant p values

## Discussion

The present retrospective study demonstrates that early missed RT (versus RT as scheduled) was an independent adverse predictor of OS in a large cohort of patients with OCSCC enrolled in an endemic betel quid chewing area. Moreover, early missed RT was independently associated with a higher occurrence of distant metastasis. Notably, late missed RT was an independent adverse predictor of local-regional control but not of OS. Taken together, our results indicate that the prognostic significance of missed sessions varies in relation to the course of RT – with early missing being independently associated with a less favorable OS.

The clinical outcomes of our patients who completed RT as scheduled were in line with those reported in previous studies [24, 25]. Conversely, growing evidence indicates that deviations from originally scheduled RT plans predict poor outcomes in patients with solid malignancies [26, 27]. A prolonged TPT has been previously associated with less favorable survival figures in head and neck malignancies [25]. A TPT > 85 days and an interval from surgery to RT initiation > 6 weeks have been related to an increased likelihood of locoregional recurrences [25, 28]. An RTT > 8 weeks was found to predict poor OS rates and a higher risk of local and distant recurrences [29] in different solid neoplasms [7, 12, 30]. Another report identified a prolonged TPT as an adverse predictor of cancer-specific survival and FFDM in patients with locally advanced laryngeal cancer [31].

Based on the available literature, it remains difficult to identify the most useful parameter for RT treatment gaps in relation to clinical outcomes. By taking advantage of a large clinical cohort of OCSCC patients treated in a homogenous manner, we deliberately used a different approach to this problem. Specifically, we investigated the prognostic impact of missed sessions according to their temporal occurrence during the course of RT. Notably, all of our patients did not have a total package time > 85 days and the time interval between surgery and RT initiation was well-balanced in the three study groups. Our findings indicate that early missed – but not late missed – RT sessions have an adverse impact on OS. Late missed RT sessions were associated with a less favorable local-regional control. While early missed sessions may exert a significant detrimental effect on survival possibly through an increased risk of distant metastases, only a trend was observed for late missed sessions. The association between early missed RT sessions and an increased occurrence of distant metastasis may be explained by the precocious effects elicited by radiation on target tissues – including alterations in immune response, cytokine signaling, and gene expression levels [32–34]. An escape of the tumor from such early effects may favor disease progression, which could account for the unfavorable prognostic significance attributable to early missed RT both in terms of OS and distant metastases. The RT-induced tissue effects elicited by initial sessions seem therefore to have a paramount prognostic significance, although the molecular underpinnings underlying this phenomenon deserve further scrutiny.

The reasons for missing RT sessions can depend on the patient (e.g., avoidance of adverse events, lack of adherence) or other factors (e.g., national holidays, machine breakdown). In general, early missed RT sessions are unlikely to be caused by treatment toxicity – whose onset generally occurs following at least 3 weeks of treatment [35]. Our current data highlight the importance of compensatory strategies when unexpected deviations from the original RT plan occur. Strategies to achieve this goal include 1) the delivery of compensatory RT sessions on weekends aimed at preserving the originally planned treatment duration, total dose, and dose per fraction, 2) an increased number of daily fractions (e.g., deliver of two fractions on Fridays with an interval of 6 h between fractions) and 3) an increased dose per fraction [36].

Our findings need to be interpreted in the context of some limitations. First, the retrospective nature of our investigation is inherently subjected to selection biases and recall biases. Second, we cannot rule out residual confounding effects due to unmeasured variables. Additionally, this is a single-institution study that may have limited external validity because it was conducted in betel quid chewing endemic area. Findings from single-center investigations are not necessarily generalizable to all patients with OCSCC because of different institutional practices and disparate patient populations. Independent confirmation of our findings is necessary before drawing more definitive conclusions.

These caveats notwithstanding, our current data indicate that early missed RT was independently associated with less favorable outcomes in patients with OCSCC who had previously undergone surgery. Efforts to maximize early adherence to RT can ultimately improve prognosis in this patient group.

## Supplementary information

**Additional file 1: Table S1.** Guidelines for postoperative treatment of oral cavity cancer in our institution.

## Data Availability

The dataset on which the study is based are available from the corresponding author upon reasonable request.

## Abbreviations

OCSCC: oral cavity squamous cell carcinoma
OS: overall survival
LRC: locoregional control
FFDM: freedom from distant metastasis
PORT: postoperative radiotherapy
3DCRT: three-dimensional conformal radiation therapy
IMRT: intensity-modulated radiation therapy
VMAT: volumetric-modulated arc therapy
RTT: radiation treatment time
TPT: treatment package time
HR: hazard ratio
CI: confidence interval

## References

[1] Bray F, Ferlay J, Soerjomataram I, Siegel RL, Torre LA, Jemal A (2018). Global cancer statistics 2018: GLOBOCAN estimates of incidence and mortality worldwide for 36 cancers in 185 countries. CA Cancer J Clin.

[2] Bureau of Health Promotion DoH, the Executive Yuan. Taiwan Cancer registry annual report, 2015, Taiwan. https://www.hpa.gov.tw/Pages/List.aspx?nodeid=269.

[3] Yao M, Chang K, Funk GF, Lu H, Tan H, Wacha J (2007). The failure patterns of oral cavity squamous cell carcinoma after intensity-modulated radiotherapy-the university of Iowa experience. Int J Radiat Oncol Biol Phys.

[4] Blanchard P, Baujat B, Holostenco V, Bourredjem A, Baey C, Bourhis J (2011). Meta-analysis of chemotherapy in head and neck cancer (MACH-NC): a comprehensive analysis by tumour site. Radiotherapy Oncol.

[5] Ooishi M, Motegi A, Kawashima M, Arahira S, Zenda S, Nakamura N (2016). Patterns of failure after postoperative intensity-modulated radiotherapy for locally advanced and recurrent head and neck cancer. Jpn J Clin Oncol.

[6] Daly ME, Le QT, Kozak MM, Maxim PG, Murphy JD, Hsu A (2011). Intensity-modulated radiotherapy for oral cavity squamous cell carcinoma: patterns of failure and predictors of local control. Int J Radiat Oncol Biol Phys.

[7] Suwinski R, Sowa A, Rutkowski T, Wydmanski J, Tarnawski R, Maciejewski B (2003). Time factor in postoperative radiotherapy: a multivariate locoregional control analysis in 868 patients. Int J Radiat Oncol Biol Phys.

[8] Su NW, Liu CJ, Leu YS, Lee JC, Chen YJ, Chang YF (2015). Prolonged radiation time and low nadir hemoglobin during postoperative concurrent chemoradiotherapy are both poor prognostic factors with synergistic effect on locally advanced head and neck cancer patients. OncoTargets Therapy.

[9] Lazarev S, Gupta V, Ghiassi-Nejad Z, Miles B, Scarborough B, Misiukiewicz KJ (2018). Premature discontinuation of curative radiation therapy: insights from head and neck irradiation. Advances Radiation Oncol.

[10] Harris JP, Chen MM, Orosco RK, Sirjani D, Divi V, Hara W (2018). Association of Survival with Shorter Time to radiation therapy after surgery for US patients with head and neck Cancer. JAMA Otolaryngol-- Head Neck Surg.

[11] Graboyes EM, Garrett-Mayer E, Ellis MA, Sharma AK, Wahlquist AE, Lentsch EJ (2017). Effect of time to initiation of postoperative radiation therapy on survival in surgically managed head and neck cancer. Cancer..

[12] Cheng YJ, Tsai MH, Chiang CJ, Tsai ST, Liu TW, Lou PJ, et al. Adjuvant radiotherapy after curative surgery for oral cavity squamous cell carcinoma and treatment effect of timing and duration on outcome-A Taiwan Cancer Registry national database analysis [published online ahead of print, 2018 Jun 14]. Cancer Med. 2018;7(7):3073–83. 10.1002/cam4.1611. 10.1002/cam4.1611 PMC6051157 29905028

[13] Robertson AG, Robertson C, Perone C, Clarke K, Dewar J, Elia MH (1998). Effect of gap length and position on results of treatment of cancer of the larynx in Scotland by radiotherapy: a linear quadratic analysis. Radiotherapy Oncol.

[14] Skladowski K, Law MG, Maciejewski B, Steel GG (1994). Planned and unplanned gaps in radiotherapy: the importance of gap position and gap duration. Radiotherapy Oncol.

[15] Tarnawski R, Fowler J, Skladowski K, Świerniak A, Suwiński R, Maciejewski B (2002). How fast is repopulation of tumor cells during the treatment gap? International journal of radiation oncology, biology. Physics..

[16] Liao C-T, Lin C-Y, Fan K-H, Wang H-M (2015). The optimal treatment modality for Taiwan Oral cavity Cancer patients-experience of a medical center. J Cancer Res Practice.

[17] Liao C-T, Lee L-Y, Hsueh C, Lin C-Y, Fan K-H, Wang H-M (2018). Pathological risk factors stratification in pN3b oral cavity squamous cell carcinoma: focus on the number of positive nodes and extranodal extension. Oral Oncol.

[18] Kang CJ, Lin CY, Yang LY, Ho TY, Lee LY, Fan KH (2015). Positive clinical impact of an additional PET/CT scan before adjuvant radiotherapy or concurrent chemoradiotherapy in patients with advanced oral cavity squamous cell carcinoma. J Nuclear Med.

[19] Tsan D-L, Lin C-Y, Kang C-J, Huang S-F, Fan K-H, Liao C-T (2012). The comparison between weekly and three-weekly cisplatin delivered concurrently with radiotherapy for patients with postoperative high-risk squamous cell carcinoma of the oral cavity. Radiat Oncol.

[20] Wang HM, Liao CT, Chang TC, Chen JS, Liaw CC, Chen IH (2004). Biweekly paclitaxel, cisplatin, tegafur, and leucovorin as neoadjuvant chemotherapy for unresectable squamous cell carcinoma of the head and neck. Cancer..

[21] Nguyen KH, Marshall L, Brown S, Neff L (2016). State-specific prevalence of current cigarette smoking and smokeless tobacco use among adults - United States, 2014. MMWR Morb Mortal Wkly Rep.

[22] Charlson ME, Pompei P, Ales KL, MacKenzie CR (1987). A new method of classifying prognostic comorbidity in longitudinal studies: development and validation. J Chronic Dis.

[23] Graboyes EM, Kompelli AR, Neskey DM, Brennan E, Nguyen S, Sterba KR (2019). Association of Treatment Delays with Survival for patients with head and neck Cancer: a systematic review. JAMA Otolaryngol-- Head Neck Surg.

[24] Cooper JS, Zhang Q, Pajak TF, Forastiere AA, Jacobs J, Saxman SB (2012). Long-term follow-up of the RTOG 9501/intergroup phase III trial: postoperative concurrent radiation therapy and chemotherapy in high-risk squamous cell carcinoma of the head and neck. Int J Radiat Oncol Biol Phys.

[25] Rosenthal DI, Mohamed ASR, Garden AS, Morrison WH, El-Naggar AK, Kamal M (2017). Final report of a prospective randomized trial to evaluate the dose-response relationship for postoperative radiation therapy and pathologic risk groups in patients with head and neck Cancer. Int J Radiat Oncol Biol Phys.

[26] Yao JJ, Zhang F, Gao TS (2019). Survival impact of radiotherapy interruption in nasopharyngeal carcinoma in the intensity-modulated radiotherapy era: a big-data intelligence platform-based analysis. Radiother Oncol.

[27] Gonzalez Ferreira JA, Jaen Olasolo J, Azinovic I, Jeremic B (2015). Effect of radiotherapy delay in overall treatment time on local control and survival in head and neck cancer: review of the literature. Reports Practical Oncol Radiotherapy.

[28] Ang KK, Trotti A, Brown BW, Garden AS, Foote RL, Morrison WH (2001). Randomized trial addressing risk features and time factors of surgery plus radiotherapy in advanced head-and-neck cancer. Int J Radiat Oncol Biol Phys.

[29] Soyfer V, Geva R, Michelson M, Inbar M, Shacham-Shmueli E, Corn BW (2014). The impact of overall radiotherapy treatment time and delay in initiation of radiotherapy on local control and distant metastases in gastric cancer. Radiation Oncol (London, England).

[30] Shaikh T, Handorf EA, Murphy CT, Mehra R, Ridge JA, Galloway TJ (2016). The impact of radiation treatment time on survival in patients with head and neck Cancer. Int J Radiat Oncol Biol Phys.

[31] Suzuki H, Terada H, Hanai N, Nishikawa D, Koide Y, Beppu S (2019). Treatment package time predicts cancer-specific survival and distant metastasis in laryngeal cancer. Oncol Lett.

[32] Sundahl N, Duprez F, Ost P, De Neve W, Mareel M (2018). Effects of radiation on the metastatic process. Mol Med.

[33] Hosseini H, Obradović MMS, Hoffmann M, Harper KL, Sosa MS, Werner-Klein M, et al. Early dissemination seeds metastasis in breast cancer. Nature. 2016;540(1476-4687 (Electronic)):552–8. 10.1038/nature20785 PMC5390864 27974799

[34] Castano Z, San Juan BP, Spiegel A, Pant A, DeCristo MJ, Laszewski T (2018). IL-1beta inflammatory response driven by primary breast cancer prevents metastasis-initiating cell colonization. Nat Cell Biol.

[35] van der Laan HP, Bijl HP, Steenbakkers RJ, van der Schaaf A, Chouvalova O, Vemer-van den Hoek JG, et al. Acute symptoms during the course of head and neck radiotherapy or chemoradiation are strong predictors of late dysphagia. Radiotherapy Oncol 2015;115(1):56–62. 10.1016/j.radonc.2015.01.019 25792467

[36] Bese NS, Hendry J, Jeremic B (2007). Effects of prolongation of overall treatment time due to unplanned interruptions during radiotherapy of different tumor sites and practical methods for compensation. Int J Radiat Oncol Biol Phys.

