# Supplementary material for: Missed radiation therapy sessions in first three weeks predict distant metastasis and less favorable outcomes in surgically treated patients with oral cavity squamous cell carcinoma
Source: Radiat Oncol. 2020 Aug 14;15:194. doi: 10.1186/s13014-020-01632-1 (PMC7427928; doi:10.1186/s13014-020-01632-1)
Supplement: Supplementary file 1 — Additional file 1: Table S1. Guidelines for postoperative treatment of oral cavity cancer in our institution. [file 13014_2020_1632_MOESM1_ESM.doc]

**Table S1.** Guidelines for postoperative treatment of oral cavity cancer in our institution

| Treatment approach | Risk factors |
| --- | --- |
| Radiotherapy (RT) | pT4 |
|  | pT3N1 (tumor >4 cm in the greatest dimension with a single affected lymph node < 3 cm) |
|  | pT1-2N1 (tumor ≤4 cm in the greatest dimension with single affected lymph node < 3 cm at neck level IV/V) |
|  | Close margins ≤ 2 mm |
|  | Poor differentiation with tumor depth ≥ 4 mm |
|  | Two minor risk factors* |
| Concurrent chemoradiation (CCRT) | Extracapsular extension |
|  | Positive margins |
|  | At least three minor risk factors* |
|  | Single ipsilateral affected lymph node ≥3 cm but <6 cm in the greatest dimension; or multiple ipsilateral affected lymph nodes (all <6 cm in the greatest dimension); or bilateral or contralateral affected lymph nodes (all <6 cm in the greatest dimension). |

*Minor risk factors: pT4, pN1, close margins ≤4 mm, poor differentiation, perineural invasion, vascular invasion, lymphatic invasion, tumor depth ≥ 10 mm
